# Rac1 augments Wnt signaling by stimulating β-catenin–lymphoid enhancer factor-1 complex assembly independent of β-catenin nuclear import

**DOI:** 10.1242/jcs.167742

**Published:** 2015-11-01

**Authors:** Cara Jamieson, Christina Lui, Mariana G. Brocardo, Estefania Martino-Echarri, Beric R. Henderson

**Affiliations:** Center for Cancer Research, The Westmead Millennium Institute for Medical Research, The University of Sydney, Westmead, New South Wales 2145, Australia

**Keywords:** β-Catenin, Rac1, Wnt signaling, Proximity ligation assay

## Abstract

β-Catenin transduces the Wnt signaling pathway and its nuclear accumulation leads to gene transactivation and cancer. Rac1 GTPase is known to stimulate β-catenin-dependent transcription of Wnt target genes and we confirmed this activity. Here we tested the recent hypothesis that Rac1 augments Wnt signaling by enhancing β-catenin nuclear import; however, we found that silencing/inhibition or up-regulation of Rac1 had no influence on nuclear accumulation of β-catenin. To better define the role of Rac1, we employed proximity ligation assays (PLA) and discovered that a significant pool of Rac1–β-catenin protein complexes redistribute from the plasma membrane to the nucleus upon Wnt or Rac1 activation. More importantly, active Rac1 was shown to stimulate the formation of nuclear β-catenin–lymphoid enhancer factor 1 (LEF-1) complexes. This regulation required Rac1-dependent phosphorylation of β-catenin at specific serines, which when mutated (S191A and S605A) reduced β-catenin binding to LEF-1 by up to 50%, as revealed by PLA and immunoprecipitation experiments. We propose that Rac1-mediated phosphorylation of β-catenin stimulates Wnt-dependent gene transactivation by enhancing β-catenin–LEF-1 complex assembly, providing new insight into the mechanism of cross-talk between Rac1 and canonical Wnt/β-catenin signaling.

## Abstract (precis)

**Summary:** Evidence is presented for a novel role of Rac1 in stimulating β-catenin–LEF-1 complex formation.

## INTRODUCTION

The canonical Wnt/β-catenin pathway plays a central role in embryonic development and adult tissue homeostasis in intestinal stem cell crypts, where it regulates the equilibrium between cell differentiation and proliferation. Wnt glycoprotein ligands attach to Frizzled transmembrane receptors and this culminates in the stabilization and nuclear translocation of β-catenin, leading to transactivation of Wnt target genes (MacDonald et al., 2009). In the absence of Wnt ligand, β-catenin is recruited into a ‘destruction complex’ consisting of adenomatous polyposis coli (APC), Axin, glycogen synthase kinase-3β (GSK-3β) and casein kinase 1 (CK1), where it is phosphorylated, ubiquitylated and degraded by the proteasome to keep β-catenin at low levels (MacDonald et al., 2009; Valenta et al., 2012). In the presence of Wnt, the destruction complex is inhibited. β-Catenin is stabilized and translocates to the nucleus where it acts as a co-activator of the T-cell factor (TCF)/lymphoid enhancer factor 1 (LEF-1) family of transcription factors, up-regulating a wide range of target genes including cyclin D1, c-myc, Axin2 and metalloproteases (MacDonald et al., 2009). Constitutive activation of Wnt signaling through gene mutations in key pathway members disturbs the finely tuned subcellular distribution and activities of β-catenin, and is linked to tumorigenesis, most notably colorectal cancer (Polakis, 2012). Indeed, increased nuclear accumulation of β-catenin is often found at the leading edge of invasive tumors (Brabletz et al., 2001).

Interestingly, cross-talk between Rac1, a small GTPase and critical regulator of actin polymerization and cell migration, and dysregulated Wnt signaling in colon adenocarcinoma cells has been reported (Esufali and Bapat, 2004; Gómez del Pulgar et al., 2007). Rac1 is a regulator of the Wnt/Jun N-terminal kinase (JNK) pathway (Coso et al., 1995; Bikkavilli et al., 2008) and other pathways including mitogen-activated protein kinase (MAPK) (Uddin et al., 2000), phosphatidylinositol 3-kinase (PI3K) and nuclear factor κB (NF-κB) (Perona et al., 1997; Murga et al., 2002). Through the activation of signaling cascades and actin cytoskeleton, Rac1 regulates intracellular adhesion, membrane ruffling, cell migration and proliferation (Sahai and Marshall, 2002). Rac1 may also modulate the Wnt/β-catenin pathway by increasing the nuclear translocation of β-catenin (Wu et al., 2008; Phelps et al., 2009). In mouse models, genetic deletion of Rac1 decreased hyperproliferation and suppressed the expansion of intestinal stem cells in APC-null crypts (Myant et al., 2013), implying that Rac1 activation contributes to Wnt-driven colorectal cancer growth. Wnt ligands have been implicated in the activation of Rac1 (Habas et al., 2003; Bikkavilli et al., 2008), and Rac1 was reported to be responsive to Wnt3a and canonical Wnt signaling (Buongiorno et al., 2008; Wu et al., 2008; Valls et al., 2012). The activation of Rac1 induces phosphorylation of β-catenin at serines 191 (S191) and 605 (S605) via the action of JNK2 kinase (Wu et al., 2008), and this was proposed to be essential for the nuclear translocation of β-catenin, suggesting that Rac1 may be required for both the stabilization and import of β-catenin to the nucleus.

The evidence for a role of Rac1 in controlling the nuclear localization of β-catenin is intriguing but controversial. A few studies reported a Rac1-dependent shift in β-catenin nuclear localization (Esufali and Bapat, 2004; Wu et al., 2008; Phelps et al., 2009), while others questioned this notion (Myant et al., 2013). Moreover, β-catenin is capable of mediating its own transit into the nucleus through direct interaction with the nuclear pore complex (Jamieson et al., 2011; Jamieson et al., 2012). Given our long-standing interest and contributions to the discovery of β-catenin nuclear transport pathways, these conflicting reports prompted us to address the mechanism by which Rac1 influences nuclear activity of β-catenin and consequently Wnt/β-catenin signaling.

## RESULTS

### Rac1 stimulates Wnt-dependent gene transactivation

**Fig. 1.**
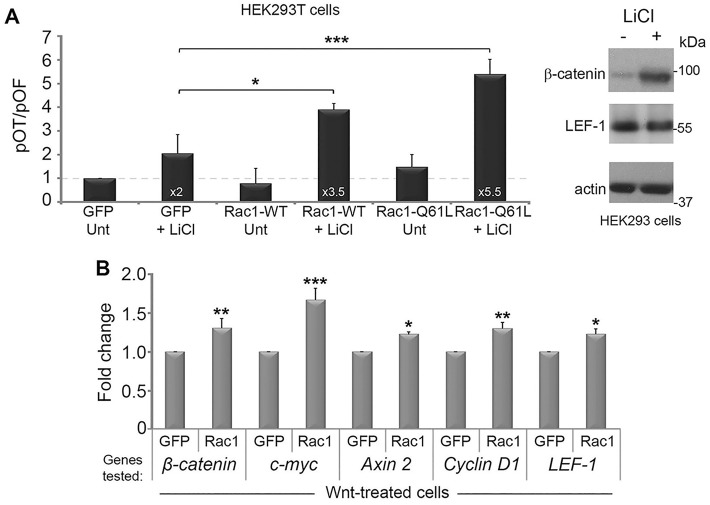
**Rac1 augments the canonical Wnt pathway.** Testing the effect of Rac1 on Wnt stimulation of LEF-1/TCF dependent promoter activity. (A) (left panel) HEK293T cells were co-transfected with plasmids encoding TOPflash/FOPflash reporters and GFP, Rac1-WT-GFP or Rac1-Q61L-GFP. At 48 h post-transfection, cells were treated with LiCl (40 mM) for 6 h to stimulate the Wnt pathway, then lysed and analyzed for LEF-1/TCF-dependent transcription by measuring luciferase activity (see Materials and Methods). Each sample was corrected for transfection efficiency by normalizing samples against GFP expression, and promoter activity displayed as a TOPflash/FOPflash (pOT/pOF) ratio. The transient expression of Rac1-WT (wild-type) and Rac1-Q61L (constitutively active) increased the positive effect of LiCl on TOPflash activity. Experiments were performed three times. Error bars represent standard deviations and statistically significant values are indicated (*P<0.01, **P<0.001, ***P<0.0001). The right-hand panel shows a western blot of total lysates from HEK293 cells treated for ±6 h with 40 mM LiCl, and confirms induction of endogenous β-catenin. LEF-1 and actin (loading control) were also stained. (B) Rac1 expression up-regulates Wnt target genes. HEK293T cells were transfected with GFP or Rac1-WT-GFP and treated with Wnt3a conditioned media (6 h). Changes in mRNA levels for β-catenin and the Wnt target genes c-myc, Axin 2, cyclin D1 and LEF-1 were determined by real-time PCR and normalized to GAPDH. Overexpression of Rac1-WT-GFP stimulated Wnt-driven gene expression by up to ∼70%. Results are presented as means±s.d. for two independent experiments. Differences between GFP alone and Rac1-WT-GFP were statistically significant where indicated. Unt, untreated.

To evaluate the sensitivity of canonical Wnt pathway induction by Rac1, we used a LEF-1/TCF reporter assay called pTOPflash. HEK293T cells were transfected with plasmids encoding GFP, Rac1-WT-GFP (wild-type) or Rac1-Q61L-GFP (constitutively active) and assayed for their responses to LiCl, a Wnt agonist, in up-regulating expression of the luciferase reporter (Fig. 1A). In the absence of LiCl, the overexpression of Rac1-WT or Rac1-Q61L was unable to significantly increase reporter activity compared with GFP alone (Fig. 1A). However, when cells were stimulated with LiCl, Rac1-WT further increased reporter activity almost 2-fold compared with GFP alone. The constitutively active form of Rac1 (Q61L) further increased activation up to 5.5-fold (Fig. 1A), suggesting that Rac1 is able to boost Wnt signaling output but only when the pathway is active. LiCl is generally a much stronger Wnt agonist than purified Wnt ligand. Therefore, to complement the above results, we performed quantitative PCR (qPCR) and evaluated β-catenin and Wnt target gene expression in cells transfected with GFP alone or Rac1-WT-GFP in the presence of Wnt. β-Catenin mRNA levels were increased by 30% and a significant Rac1-dependent increase in additional Wnt target genes including c-myc (70%), Axin 2 (20%), cyclin D1 (30%) and LEF-1 (20%) was also observed (Fig. 1B). Taken together, these data verify the ability of Rac1 to positively augment active Wnt signaling.

### Rac1 does not stimulate β-catenin nuclear localization in the absence or presence of LiCl

**Fig. 2.**
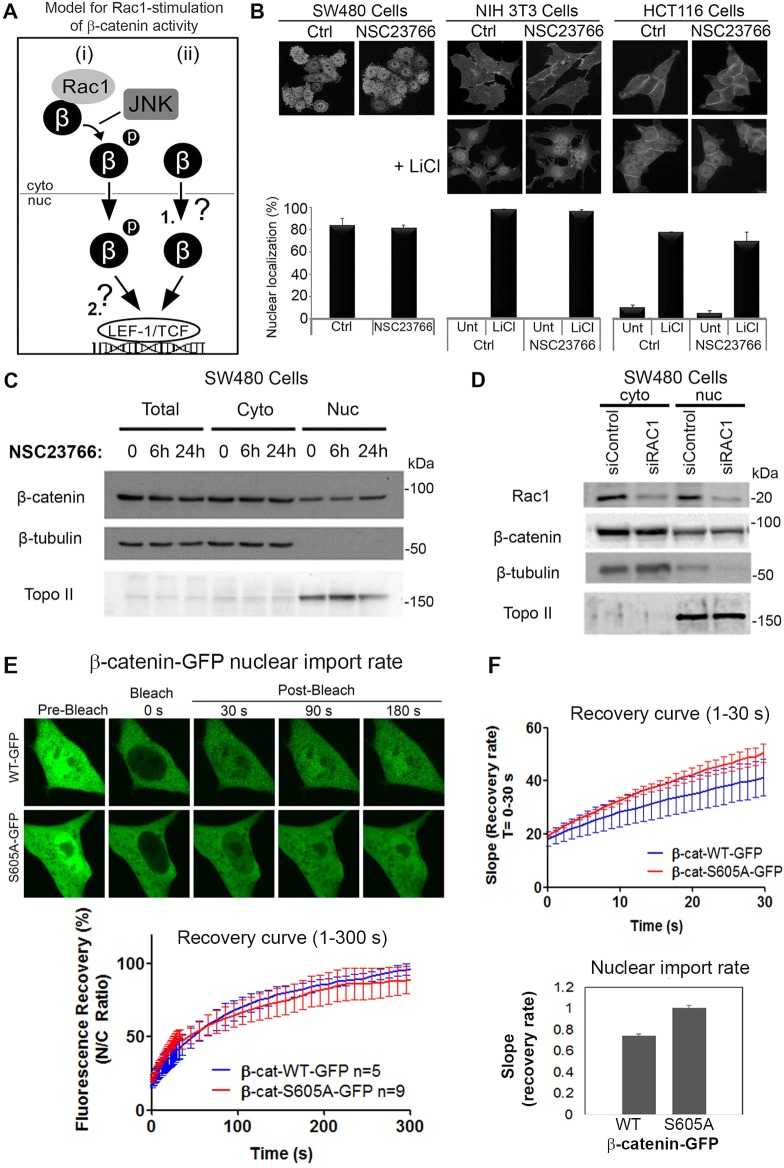
**Blocking Rac1 activity or expression does not alter β**-**catenin nuclear localization.** (A) Two likely mechanisms by which Rac1 can stimulate Wnt/β-catenin gene transactivation are shown: (i) Rac1-mediated phosphorylation of β-catenin drives nuclear import and accumulation of β-catenin (Wu et al., 2008; Phelps et al., 2009), and (ii) unphosphorylated β-catenin can enter the nucleus (1) and Rac1-mediated phosphorylation of β-catenin stabilizes its interaction with LEF-1 to drive transactivation (2). (B) To test the first model, different cell lines were treated for 6 h with 50 µM NSC23766 (Rac1 inhibitor) in the absence and presence of 40 mM LiCl. Cells were then fixed and stained for β-catenin, then analyzed by immunofluorescence microscopy. Representative cell images are shown. Two hundred cells were scored for nuclear localization of β-catenin and the data are plotted as means±s.d. from two independent experiments. The Rac1 inhibitor had no significant effect. (C) SW480 cells were treated with NSC23766 (50 µM) for 6 or 24 h, after which cells were lysed and β-catenin protein levels analyzed by western blotting. Blots were probed for anti-β-catenin, β-tubulin (cytoplasmic loading control; cyto) and Topoisomerase II (Topo II, nuclear loading control; nuc). (D) Rac1 knockdown did not affect β-catenin distribution. SW480 cells were transfected with Rac1 or control siRNAs for 72 h. Cells were then lysed and subjected to western blotting. Blots were probed for anti-Rac1, β-catenin, β-tubulin and Topo II. (E) pβ-catenin-WT-GFP and pβ-catenin-S605A-GFP were expressed in NIH3T3 cells and compared for nuclear import by FRAP. Nuclear fluorescence was bleached and recovery measured over 300 s (see cell images and recovery curve). (F) Analysis of the first 30 s post-bleach revealed that the S605A mutation did not inhibit nuclear import of β-catenin.

There are two likely pathways (but not necessarily the only ones) by which Rac1 might increase Wnt/β-catenin-dependent gene activation as shown above. The favored mechanism currently proposed in the literature is that Rac1-mediated phosphorylation of β-catenin drives the nuclear translocation and accumulation of β-catenin (Schlessinger et al., 2009; Valenta et al., 2012). However, it is also possible that Rac1 stimulates β-catenin nuclear transactivation function by stabilizing the interaction between β-catenin and LEF-1 (summarized in Fig. 2A). To distinguish between these possibilities, we first performed a rigorous examination of β-catenin subcellular distribution following alteration of Rac1 activity and/or expression in multiple cell lines.

To test whether Rac1 can promote the nuclear translocation of β-catenin, we inhibited Rac1 activity and analyzed β-catenin subcellular distribution in human colon cancer cell lines with mutated β-catenin (SW480, HCT116) and fibroblastic (NIH 3T3) cell lines using an Olympus FV1000 confocal microscope. SW480, NIH 3T3 and HCT116 cells were treated for 6 h with 50 µM of the Rac1 inhibitor, NSC23766, which inhibits Rac1 by trapping it in a predominantly GDP (inactive) form, and then cells were fixed and immunostained for microscopy (Fig. 2B). The Rac1 inhibitor did not affect the nucleo-cytoplasmic distribution of β-catenin in any of the three cell lines tested. Additionally, NIH 3T3 and HCT116 cells were pre-treated with NSC23766 for 1 h prior to the addition of LiCl, used to stimulate the Wnt pathway through its inhibition of GSK-3β (Fig. 2B). As expected, the LiCl treatment induced clear nuclear accumulation of β-catenin; however, NSC23766 was unable to prevent β-catenin translocating to the nucleus. This effect appeared to be unrelated to whether the Wnt pathway was intact (NIH 3T3) or perturbed by gene mutations (HCT116, SW480) (Fig. 2B). We then applied a cell fractionation protocol, which separated the nuclear compartment from the cytoplasm of SW480 cells. Under control conditions, β-catenin was detected in both the nuclear and cytosol fractions (Fig. 2C). Upon treatment with NSC23766, we saw no change in β-catenin cellular distribution or protein levels compared with control at both the 6 and 24 h time points (Fig. 2C). The drug was made fresh for each application and drug efficacy was confirmed by observing its ability to block Rac1-dependent membrane ruffle formation (see Fig. S1A). Similar results were observed using a different Rac1 inhibitor, EHT 1864 (Fig. S1B). Furthermore, even when Rac1 expression was silenced by siRNA treatment we observed no change in β-catenin nuclear/cytoplasmic distribution or protein levels, as revealed by cell fractionation and western blot analysis (Fig. 2D). Together, these data imply that Rac1 inhibition does not affect the nucleo-cytoplasmic distribution or cellular levels of β-catenin in commonly used cell lines of different origin.

### Mutations in Rac1-sensitive residues S191 and S605 do not alter β-catenin nuclear import rate or localization

The activation of Rac1 was reported to induce the JNK kinase-dependent phosphorylation of β-catenin at S191 and S605, which is essential for Rac1 regulation of β-catenin (Wu et al., 2008). Mutation of the S191 and S605 residues was claimed to impair the nuclear translocation of β-catenin (Wu et al., 2008; Phelps et al., 2009). To address the importance of these specific Rac1-sensitive serine residues, SW480 cells were transfected with several β-catenin constructs containing inactivating (S191A, S605A) or hyperactivating phosphomimetic (S191D, S605D) mutations in these sites (Fig. S1C). Fluorescence confocal microscopy and visual scoring of the immunostained cells revealed that all mutants showed predominantly a nucleo-cytoplasmic distribution similar to that observed for WT-β-catenin (Fig. S1C,D). This result was striking given that SW480 cells express a mutant form of APC (amino acids 1–1337), which has been implicated as a prerequisite for Rac1-dependent nuclear targeting of β-catenin (Esufali and Bapat, 2004). More importantly, we have directly tested the effect of the S605A mutation on nuclear import rate. GFP-tagged β-catenin wild-type and S605A mutants were expressed in NIH 3T3 cells and their rate of nuclear entry compared after photobleaching the nucleus (Fig. 2E). The wild-type β-catenin entered the nucleus at a similar rate to that previously reported (Jamieson et al., 2011), and unexpectedly the S605A mutant actually entered slightly faster than the wild-type sequence (Fig. 2F). We conclude that the mutation of Rac1 target sites does not diminish the rate of nuclear import or extent of nuclear accumulation of β-catenin.

### Overexpression of Rac1 does not affect the subcellular distribution of endogenous β-catenin

**Fig. 3.**
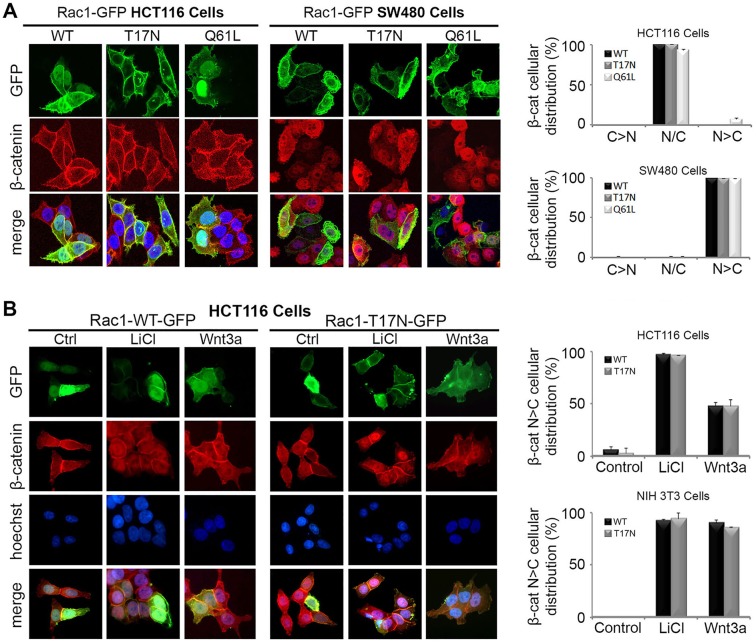
**Overexpression of Rac1 does not affect nuclear localization of endogenous β**-**catenin in HCT116, SW480 or NIH 3T3 cells.** (A) HCT116 and SW480 colorectal cancer cells were transfected with plasmids for GFP-tagged WT (wild-type), T17N (dominant negative) or Q61L (constitutively active) Rac1. Immunostaining with β-catenin antibody and microscopy of cells revealed that endogenous β-catenin (red) remains nuclear/cytoplasmic (N/C) in distribution regardless of Rac1-GFP (green) localization. The cytoplasmic (C) and nuclear (N) distribution of β-catenin is shown in the bar graphs (C>N, N/C, N>C) for each Rac1 construct. (B) Next, a dominant negative mutant form of Rac1 (T17N) was tested for its ability to block β-catenin nuclear accumulation in Wnt-stimulated HCT116 and NIH 3T3 cells. Cells were transfected with pRac1-WT-GFP or pRac1-T17N-GFP, treated with 40 mM LiCl or Wnt3a conditioned media for 6 h and then stained for GFP (green), β-catenin (red) and DNA (blue). Typical microscopy images of transfected HCT116 cells are shown. Column graph displays the cellular distribution (N>C) of endogenous β-catenin in Rac1-WT or T17N transfected cells in the absence and presence of LiCl/Wnt3a in HCT116 and NIH 3T3 cells.

An earlier study suggested that Rac1 requires constitutively activated Wnt signaling and/or elevated β-catenin levels in order to further augment Wnt signaling (Esufali and Bapat, 2004). To investigate this, plasmid constructs encoding wild-type (WT), dominant negative (T17N) and constitutively active (Q61L) Rac1 were transiently expressed in HCT116 and SW480 colon cancer cells, which have mutations in β-catenin and APC, respectively, leading to deregulated canonical Wnt signaling. The Q61L-Rac1 mutant exists in a constitutively active GTP-bound form, while the T17N dominant negative adopts an inactive GDP-bound form. As shown in Fig. 3A, ectopic Rac1-WT exhibited a uniform nucleo-cytoplasmic distribution in both cell lines, while the Rac1-T17N dominant negative mutant was more cytoplasmic/membrane localized, and the Rac1-Q61L active mutant displayed a dominantly nuclear expression pattern. In HCT116 cells, endogenous levels of β-catenin are raised and evenly distributed throughout the cell, with a large proportion retained at the cell membrane. This distribution was unchanged after expression of all three Rac1-GFP constructs (Fig. 3A). Similarly, in SW480 cells that display high levels of nuclear β-catenin there was no change in β-catenin cellular distribution in Rac1 transfected cells (Fig. 3A).

Next, Rac1-WT (wild-type) and T17N mutants were transiently expressed in HCT116 cells in the absence and presence of LiCl or Wnt3a conditioned medium (Fig. 3B). According to a previous report, overexpression of Rac1-T17N can prevent the nuclear induction of β-catenin after Wnt signaling (Wu et al., 2008). However, as shown in Fig. 3B, neither wild-type nor dominant negative Rac1 altered the nuclear induction of β-catenin after LiCl or Wnt3a treatment. Similar results were observed in NIH 3T3 cells (Fig. 3B, column graph). These data imply that the dominant negative form of Rac1 is not sufficient to inhibit the nuclear induction and accumulation of β-catenin upon Wnt activation.

### Activation of Rac1 causes dissociation of Rac1–β-catenin complexes from the plasma membrane

If Rac1 does not stimulate nuclear import of β-catenin, then how does it augment the Wnt pathway? We speculated that the site of Rac1–β-catenin complex assembly might provide some insights. To address this we investigated the subcellular locations at which Rac1–β-catenin complexes form, and asked whether this might change in response to Rac1 or Wnt activation. These issues have not previously been addressed.

**Fig. 4.**
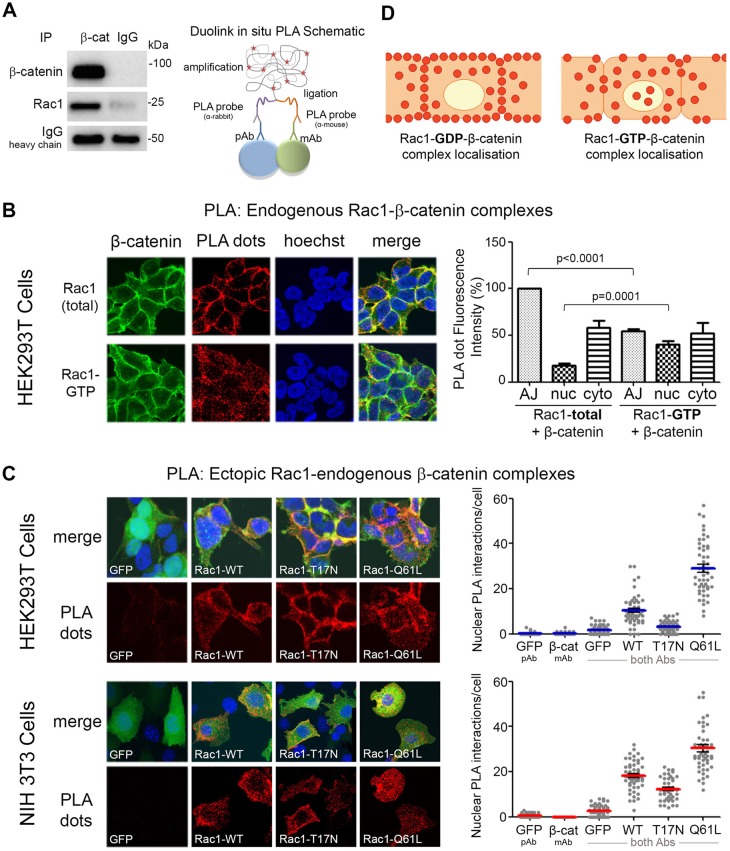
**Activation of Rac1 causes Rac1**–**β**-**catenin complexes to move from the plasma membrane to the nucleus.** (A) Left panel: IP of endogenous β-catenin detects association with Rac1 in HEK293 cell lysate; right panel: schematic diagram illustrates the steps involved with the Duolink *in situ* PLA. The process consists of primary antibodies and PLA probes binding to the target proteins (e.g. β-catenin and Rac1), hybridization and ligation followed by rolling circle amplification. (B) HEK293T cells were stained with primary antibodies for either endogenous total Rac1 or active Rac1 (GTP) and β-catenin, processed for detection of Rac1–β-catenin complexes by PLA technique and imaged using confocal microscopy (see Materials and Methods). Each red dot represents a single interaction between Rac1 and β-catenin. Nuclei were stained with Hoechst (blue) and β-catenin cellular staining is shown in green. Column graph displays the fluorescence intensity of the PLA red dots at the adherens junctions (AJ), nucleus (nuc) or cytosol (cyto) for Rac1(total)–β-catenin and Rac1(GTP)–β-catenin complexes. There was a significant increase in active Rac1–β-catenin complexes in the nucleus, compared with total Rac1–β-catenin complexes, correlating with a reduction in complexes at the adherens junctions. Control images can be found in Fig. S2A. (C) HEK293T and NIH 3T3 cells were transfected with plasmids for GFP-tagged WT (wild-type), T17N or Q61L Rac1 before being stained with anti-GFP and anti-β-catenin antibodies to detect interactions between Rac1 and β-catenin using the PLA. Cells were then stained for β-catenin (green) and DNA (blue), and the PLA-positive red dots each represent an amplified Rac1-GFP/β-catenin interaction complex. For the controls, only one primary antibody (β-catenin or GFP) was added to each well (Fig. S2B). The dot plot represents the number of positive nuclear PLA signals per cell for both the single antibody controls (GFP pAb or β-catenin mAb) or both antibodies for each Rac1 construct. The dot plot is representative of two independent experiments each containing 50 cells scored from *z*-stack confocal microscopy images. (D) Schematic diagram illustrating Rac1–β-catenin complex cellular distribution. When Rac1 is predominantly in its inactive form, Rac1(GDP)–β-catenin complexes are strongly located at the membrane and adherens junctions. Alternatively, when Rac1 is active, Rac1(GTP)–β-catenin complexes displayed a more nuclear-cytoplasmic distribution pattern.

We first detected endogenous Rac1–β-catenin complexes in HEK293 cell total lysates by immunoprecipitation (IP) (Fig. 4A, left panel). We were able to identify Rac1–β-catenin complexes in cytoplasmic fractions, and to some extent also in nuclear fractions by IP (see Fig. S2A); however, a more sensitive and consistent approach was required. To more effectively investigate Rac1–β-catenin complex localization we used an *in situ* microscopy approach using a proximity ligation assay (PLA). PLA is an antibody-based method in which two proteins are immunolabeled: first with primary antibodies and then with secondary antibodies conjugated to complementary oligonucleotides (Söderberg et al., 2008). When the two antibody molecules are in close proximity, the complementary DNA strands can be ligated, amplified and visualized as distinct fluorescent puncta (outlined in Fig. 4A, right panel). For this assay, cells were fixed and subjected to PLA using rabbit anti-β-catenin and mouse anti-Rac1 (total and active) antibodies with the Duolink kit (see Materials and Methods). Endogenous complexes between total Rac1–β-catenin and active Rac1–β-catenin were observed by confocal microscopy as red dots (Fig. 4B) and the controls were clean (Fig. S2B,C). Positive interactions were observed for both types of complex but their distribution patterns were significantly different (Fig. 4B). Interestingly, total Rac1–β-catenin complexes were mainly located at the plasma membrane including the adherens junctions, whereas active Rac1–β-catenin complexes preferentially located to the nuclear-cytoplasmic region. To further investigate this phenomenon we transfected NIH 3T3 fibroblasts and HEK 293T cells with different Rac1 constructs and compared the resulting distribution patterns of the Rac1–β-catenin complexes. As shown in Fig. 4C, cells transfected with dominant negative Rac1 (T17N) formed complexes with endogenous β-catenin preferentially at the membrane, while cells transfected with the constitutively active form of Rac1 (Q61L) displayed a shift in complexes with β-catenin to the cytosol and nucleus. Indeed, quantification of cell image *z*-stacks shows that the number of nuclear PLA interactions between T17N-Rac1 and β-catenin was the lowest of all three Rac1 constructs tested. The number of nuclear interactions increased with expression of WT-Rac1 and again with the active Rac1 (Fig. 4C). These data corroborate the differential localization patterns seen in Fig. 4B, and the shift in distribution is summarized in Fig. 4D.

**Fig. 5.**
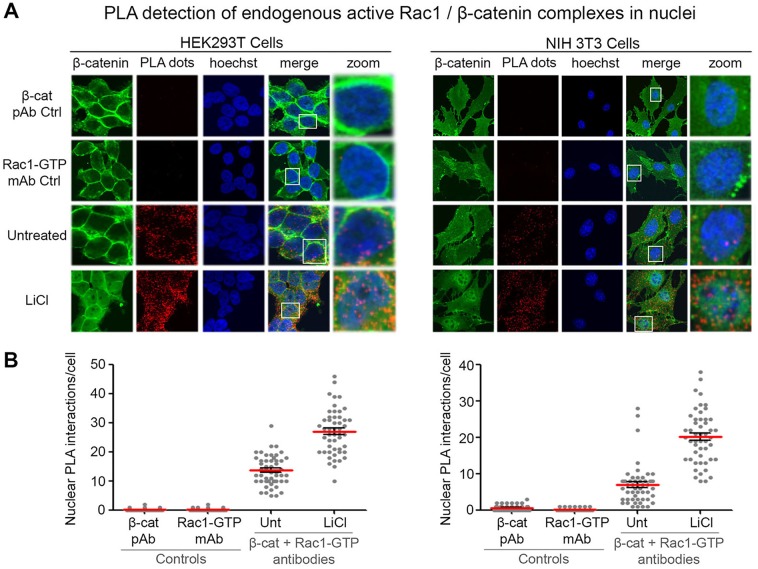
**The Wnt agonist LiCl stimulates formation of endogenous active Rac1**–**β-catenin complexes in the cytoplasm and nucleus.** *In situ* PLA in HEK293T and NIH 3T3 cells after Wnt stimulation with LiCl. (A) Cells were treated with 40 mM LiCl for 6 h and antibodies against active Rac1 and β-catenin were used to detect active Rac1–β-catenin complexes. The primary antibodies Rac1-GTP and β-catenin were used alone as controls (top two rows). Cells were counterstained with anti-β-catenin (green) and Hoechst (blue). (B) Positive PLA signals (red dots) were counted from *z*-stack confocal images and the number of dots in the nuclei of cells is presented in the dot plots for each cell line. Fifty nuclei per experiment were counted over two independent experiments and a representative plot is displayed. Unt, untreated.

Next, to determine if Wnt stimulation affected the distribution of Rac1–β-catenin complexes, the PLA was repeated in the presence and absence of the Wnt agonist LiCl in HEK293T and NIH 3T3 cells. Cells were fixed and subjected to the Duolink PLA method using anti-β-catenin and anti-active Rac1 antibodies. In the untreated sample we observed a nucleo-cytoplasmic distribution of active Rac1–β-catenin complexes (Fig. 5); however, in the presence of LiCl we detected both an increase in total number of interactions per cell and a further increase in nuclear-localized complexes, which was also observed using the total Rac1 antibody (Fig. S2D,E). This increase in nuclear complexes did not result from a direct effect of Wnt on Rac1 expression or distribution (Fig. S3A,B) but appeared to result indirectly from stabilization and increased levels of β-catenin.

### Rac1 stimulates β-catenin–LEF-1 complex formation in the nucleus

**Fig. 6.**
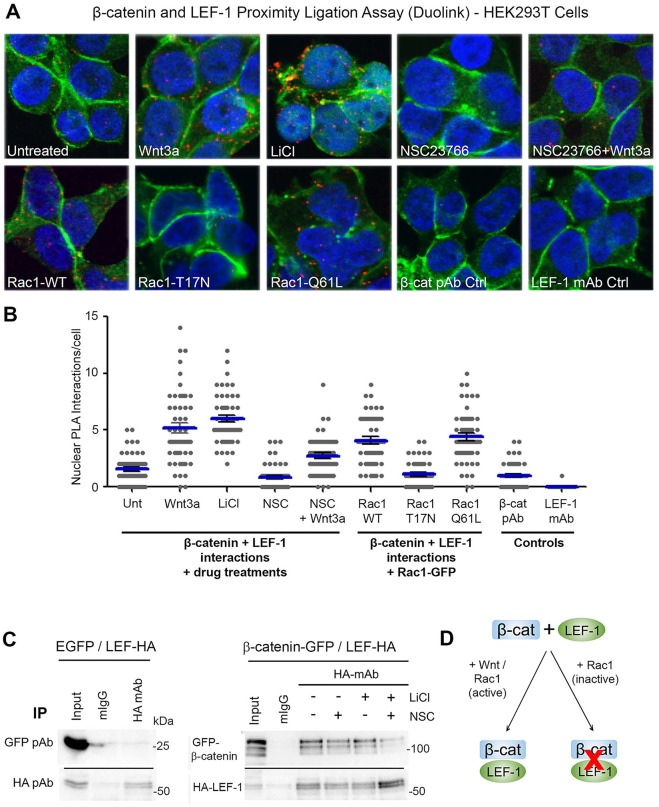
**Rac1 stimulates the formation of endogenous β**-**catenin**–**LEF-1 complexes.** (A) HEK293T cells were stained with primary antibodies and processed for detection of interactions between endogenous β-catenin and LEF-1 using the proximity ligation assay (PLA) Duolink. As shown in the cell images, cells were either transfected with Rac1 constructs (WT, T17N or Q61L) or treated with Wnt3a conditioned media, 40 mM LiCl, 50 µM NSC23766 or 50 µM NSC23766+Wnt3a for 6 h before being fixed and subjected to Duolink assay. The assay was performed using both anti-β-catenin and anti-LEF-1 antibodies (see Materials and Methods for details). For the control wells, only one primary antibody (β-catenin or LEF-1) was added. Cells were then stained for β-catenin (green) and the PLA-positive red dots detected each represent an amplified β-catenin/LEF-1 interaction complex. (B) The PLA signals were counted and the number of dots in the nucleus per cell from single slice confocal images is presented in the plot. One hundred nuclei from two independent experiments were scored. (C) GFP, GFP-β-catenin and HA-tagged LEF1 were transiently expressed in HEK293 cells and total lysates used for IP by anti-HA antibody. The IP shows detection of LEF-1–β-catenin complexes in cells untreated or treated for 6 h with drug. (D) Summary diagram showing that the interaction between β-catenin and LEF-1 is stimulated in the presence of Wnt and active Rac1, and inhibited in the presence of inactive Rac1.

We showed above that Rac1 activation and Wnt both stimulate the formation of active Rac1–β-catenin complexes in the cytoplasm and nucleus. Next, we tested the hypothesis that nuclear Rac1 can influence the interaction between β-catenin and transcription factor LEF-1. HEK293T cells were transfected with plasmids expressing Rac1 (WT, T17N or Q61L) and treated for 6 h with: (i) a Wnt stimulus (Wnt3a conditioned media or 40 mM LiCl), (ii) a Rac1 inhibitor (50 µM NSC23766), or (iii) combination of both 50 µM NSC23766+Wnt3a. Cells were then fixed and subjected to Duolink PLA using rabbit anti-β-catenin and mouse anti-LEF-1 antibodies, and endogenous complexes between β-catenin and LEF-1 were then detected as red dots by fluorescent microscopy (see cell images in Fig. 6A). In untransfected cells with no treatment, a low level of endogenous β-catenin–LEF-1 complexes (average of ∼1.5 to 2 dots per nucleus) was observed. Treatment of cells with Wnt3a or LiCl stimulated the number of positive protein interactions >3-fold (Fig. 6B). Similarly, the transient expression of WT-Rac1 or constitutively active (Q61L)-Rac1 caused a significant increase in nuclear β-catenin–LEF-1 interactions relative to control (Fig. 6B). Conversely, overexpression of dominant negative Rac1 (T17N) had no effect on β-catenin–LEF-1 complex formation, underscoring the specificity of the results seen with the WT- and Q61L-mutant Rac. Moreover, treatment with the Rac1 inhibitor NSC23766 resulted in a marked reduction in interactions between β-catenin and LEF-1 in the nuclei of Wnt-treated cells (Fig. 6B). Importantly, we were able to show by IP that the Rac1 inhibitor reduced formation of ectopic LEF-1–β-catenin complexes both before and after LiCl treatment (Fig. 6C; Fig. S3D–F). The reduction observed in LEF-1–β-catenin complex formation was not due to altered LEF-1 levels (Fig. 6C; Fig. S4A). Note that we were unable to detect an interaction between LEF-1 and Rac1 (Fig. S3C), so it is unlikely that all three proteins are in complex at chromatin. These results, when considered together, suggest that active Rac1 can stimulate complex formation between β-catenin and LEF-1 and contributes to the positive effect of Wnt on the assembly of β-catenin–LEF-1 complexes in cells (Fig. 6B,D).

### Mutational inactivation of Rac1-regulatory sites of β-catenin cause a reduction in its binding to LEF-1

**Fig. 7.**
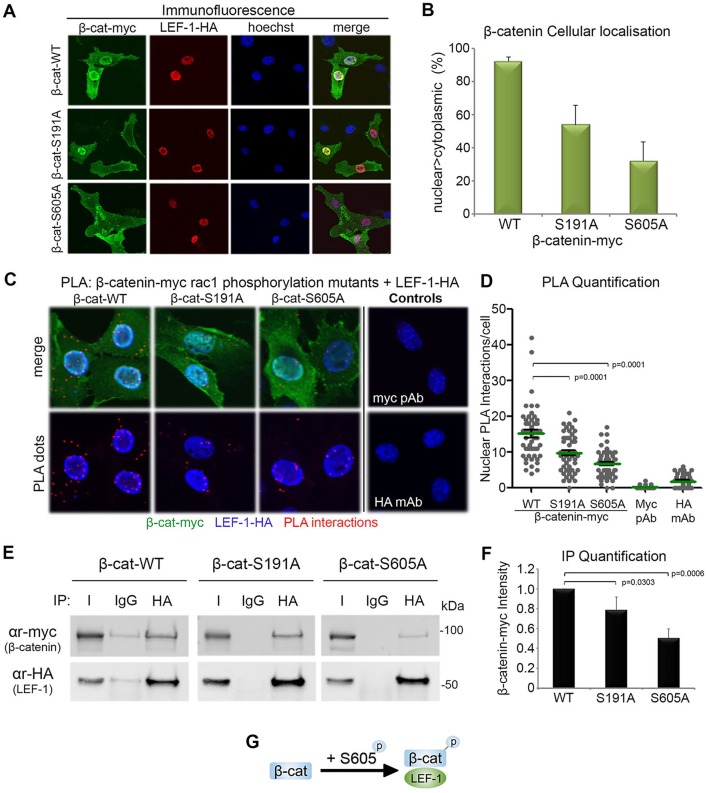
**Mutation of the Rac1**-**regulated phosphorylation sites of β**-**catenin reduce its binding to LEF-1.** Overexpression of pHA-LEF-1 anchors β-catenin to chromatin and stabilizes it in the nucleus of NIH 3T3 cells (Henderson et al., 2002). (A) We used this ability of LEF-1 to anchor β-catenin in the nucleus to first compare LEF-1 binding efficiency of wild-type and Rac1-regulatory site mutants (S191 and S605) of β-catenin. NIH 3T3 cells were co-transfected with plasmids encoding myc-tagged WT, S191A or S605A β-catenin and pHA-LEF-1 before being fixed and stained for α-myc (green: β-catenin), α-HA (red: LEF-1) and DNA (blue: Hoechst dye,). LEF-1 is seen to drag WT-β-catenin into the nuclei of cells more efficiently than it does for β-catenin-S191A and S605A mutants. (B) Cells were scored for nuclear>cytoplasmic staining of ectopic β-catenin in the LEF-1-transfected cells. (C) NIH 3T3 cells were co-transfected with myc-tagged β-catenin constructs (WT, S191A or S605A) and pHA-LEF-1 and subjected to PLA. α-myc and α-HA antibodies were used to detect the interaction between β-catenin and LEF-1. A significant reduction in nuclear interactions between the β-catenin mutants (S191A and S605A) and LEF-1-HA can be seen. (D) A dot plot shows the number of nuclear interactions between β-catenin and LEF-1 counted per cell. One hundred nuclei over two independent experiments were scored from *z*-stack confocal microscopy images and a representative graph is shown. (E) pLEF-1-HA was co-transfected with pβ-catenin-myc constructs (WT, S191A or S605A) in NIH 3T3 cells. Cell lysates were subjected to immunoprecipitation using HA mAb or isotype control IgG antibody. The blot was probed with anti-HA and myc antibodies and shows a reduction in β-catenin-phosphorylation mutant binding to LEF-1 compared with WT-β-catenin. (F) Column graph showing significant reduction in β-catenin mutant band intensity compared with wild-type analyzed by densitometry over three independent experiments. (G) Schematic diagram showing the phosphorylation of β-catenin at S605 (Rac1 mediated) increases the interaction between β-catenin and LEF-1.

To gain further insight into the effect of Rac1 on the interaction between β-catenin and LEF-1 we analyzed the consequences of mutating the Rac1-sensitive β-catenin serine residues S191 and S605 on binding of LEF-1. NIH 3T3 cells were co-transfected with myc-tagged β-catenin constructs (WT, S191A and S605A) and pHA-LEF-1. When analyzed by fluorescence microscopy, we observed that ectopically expressed LEF-1 (stained red) caused a stable retention of WT-β-catenin (stained green) in the nuclei of >90% of transfected cells (see images in Fig. 7A). In contrast, those forms of β-catenin with mutation of the Rac1-regulated phosphorylation sites (i.e. S191A and S605A) were less influenced by LEF-1 and displayed a substantially reduced nuclear staining pattern (down to 55 and 35%) as shown in Fig. 7B. This result was not due to a defect in the ability of S191A and S605A mutants to enter the nucleus, as they displayed a nuclear shift in response to LiCl treatment of NIH 3T3 cells similar to that of wild-type β-catenin (see Fig. S4B,C). This suggested that S191 and S605 may contribute to binding of LEF-1. To compare the interaction between these Rac1-specific mutants of β-catenin and LEF-1, a PLA was used. NIH 3T3 cells were co-transfected with myc-tagged β-catenin constructs (WT, S191A and S605A) and HA-LEF-1, and then anti-myc and anti-HA antibodies were used for the assay. To confirm specificity of the *in situ* assay each antibody was used in isolation to ensure that no cross-reactivity was present (Fig. 7C, control panel). As revealed in Fig. 7C, expression of WT-β-catenin exhibited the highest number of interactions with LEF-1 in nuclei, and this was significantly reduced for the S191A mutant (*P*<0.0001) and even more substantially disrupted by the S605A mutation (*P*<0.0001) (Fig. 7C,D). It is interesting to note that mutation of S605 had a more detrimental impact on LEF-1 binding. This experiment was also performed with endogenous LEF-1 with similar results (C. Jamieson, unpublished data).

We next used immunoprecipitation assays to measure binding efficacy between myc-tagged forms of β-catenin and LEF-1, using an anti-HA antibody to pull down ectopic HA-LEF-1. Remarkably, the trends observed in the immunoprecipitation experiments (Fig. 7E) were almost identical to that seen with the PLA (Fig. 7D). β-Catenin-WT showed the strongest interaction, and relative to this the S191A mutation caused a small (∼20%) reduction in binding, whereas the S605A mutation caused a ∼50% reduction in β-catenin–LEF-1 binding (Fig. 7F). Collectively, these data suggest that Rac1-mediated phosphorylation of β-catenin at S605 and S191 enhances complex formation between β-catenin and LEF-1, with S605 playing a more critical role (Fig. 7G).

## DISCUSSION

**Fig. 8.**
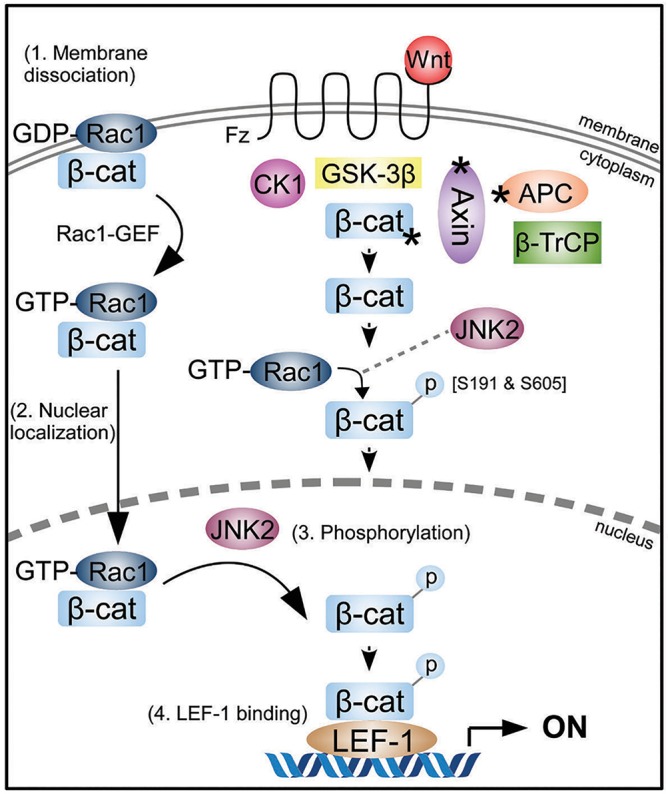
**Rac1 activation and Wnt synergistically enhance β-catenin transactivation in the canonical Wnt pathway.** In Wnt stimulated cells or in cancer when there are mutations in key pathway components (*Axin, APC, β-catenin) β-catenin is driven into the nuclei of cells to transactivate Wnt target genes in co-operation with LEF-1/TCF transcription factors. In addition to Wnt, active Rac1 augments the transcription of Wnt target genes through the following steps: (1) loss of β-catenin membrane retention after Rac1 activation; (2) increased nuclear localization of active Rac1–β-catenin complexes; (3) phosphorylation of β-catenin at S191 and S605 by JNK2, leading to (4) increased binding of phosphorylated β-catenin to the transcription factor LEF-1 and enhanced transactivation. It is possible that phosphorylation of β-catenin at S191 and S605 could occur in either nucleus or cytoplasm.

In this study we demonstrate that Rac1 does not affect the nuclear-cytoplasmic distribution of β-catenin in the absence or presence of Wnt, contrary to current theories (Wu et al., 2008; Phelps et al., 2009). Our findings instead suggest that Rac1 enhances the interaction between β-catenin and LEF-1, providing a plausible new explanation for the role of Rac1 in augmenting transactivation of Wnt target genes. The extensive cell imaging of PLA data identified specific subcellular distribution patterns of Rac1–β-catenin complexes, which varied with Rac1 activation status, and indicate that both activation of Rac1 and Wnt stimulation drive the nuclear accumulation of a pool of β-catenin–Rac1 complexes. We propose that this stimulates binding of β-catenin to LEF-1, leading to enhanced Wnt signaling responses. Indeed, increased levels of active Rac1 (GTP bound) were previously found in murine intestines where Wnt is constitutively activated due to loss of APC (Malliri et al., 2006; Myant et al., 2013). Our model (Fig. 8) details the proposed steps for Rac1 involvement in canonical Wnt signaling: (1) activation (GTP-loading) of Rac1 reduces its association with the plasma membrane and causes active Rac1–β-catenin complexes to dissociate from the membrane, (2) these active Rac1–β-catenin complexes translocate to the cytoplasm and nucleus, (3) active Rac1 mediates the phosphorylation of β-catenin at S191 and S605 via JNK2 kinase in the cytoplasm and possibly the nucleus, and (4) the newly phosphorylated β-catenin preferentially binds to LEF-1 to boost gene transactivation.

### Rac1 does not affect the nuclear localization of β-catenin

Phosphorylation of β-catenin is a key mechanism for regulating its stability and subcellular localization (Valenta et al., 2012; Gao et al., 2014). Indeed, β-catenin residues S191 and S605 were found to be phosphorylated by JNK2 in the cytoplasm in response to Rac1 activity. These residues were claimed to be necessary for nuclear translocation of β-catenin (Wu et al., 2008) and previous studies indicated a cytoplasmic distribution for S191A and S605A β-catenin (Wu et al., 2008; Phelps et al., 2009). In contrast, we showed by confocal cell imaging that there was no significant effect of these mutations on either the nuclear distribution (Fig. S1C) or nuclear import rate (Fig. 2E,F) of β-catenin. β-Catenin phospho-mimic variants (serine to aspartate; S191D and S605D) were also evaluated and had no effect on nuclear distribution. Furthermore, Rac1 silencing or inactivation did not affect the nucleo-cytoplasmic levels or distribution of endogenous β-catenin in SW480 colorectal cells (Fig. 2), a finding consistent with suggestions made by the Sansom laboratory (Myant et al., 2013).

Rac1 contains a nuclear localization signal (Lanning et al., 2004) and displays rapid shuttling kinetics *in vivo* (Johnson et al., 2013), suggesting that Rac1 may translocate to the nucleus with β-catenin to influence transcriptional activation. Indeed, β-catenin and Rac1 have been reported to form complexes in the nuclei of HEK293 cells (Esufali et al., 2007) and HCT116 cells (Buongiorno et al., 2008). Interestingly, Rac1 has been shown to enhance the nuclear accumulation of protein complexes comprising other armadillo domain-containing proteins such as SmgGDS and p120 catenin (Lanning et al., 2003), consistent with the previous assumption that Rac1 regulates nuclear accumulation of β-catenin. However, in this study, we reproduced many of the key experiments previously reported but with quite different results (Fig. 3). Overexpression of Rac1-Q61L, a constitutively active mutant, was unable to induce nuclear uptake of β-catenin in HCT116 cells despite dysregulated Wnt signaling in these cells due to deletion of codon 45 in one allele of β-catenin (Sparks et al., 1998), which has been suggested as a prerequisite for Rac1 augmentation of the Wnt pathway (Esufali and Bapat, 2004). Conversely, dominant negative Rac1 failed to block nuclear entry of β-catenin under any condition, leading us to conclude that Rac1 does not modulate the nuclear translocation of β-catenin in response to Wnt, at least in those cell lines studied.

Despite lack of evidence supporting a Rac1-mediated import of β-catenin, Rac1 was able to significantly augment TCF/LEF-1-mediated transcription (Fig. 1A) and up-regulate mRNA transcripts of well-characterized Wnt target genes including cyclin D1, c-myc, LEF-1 and Axin2 (Fig. 1B) (He et al., 1998; Shtutman et al., 1999; Hovanes et al., 2001; Jho et al., 2002). This was observed only when Wnt signaling was active, in accordance with a prior study where Rac1-WT alone did not affect TOPflash activity (Buongiorno et al., 2008) and an earlier report where mouse embryos lacking JNK2 did not exhibit the gastrulation defect normally associated with loss of canonical Wnt signaling (Kuan et al., 1999). TCF/β-catenin strongly activates c-jun transcription in colon cancer cells (Mann et al., 1999; Nateri et al., 2005) suggesting that Rac1 has the ability to up-regulate c-myc transcription via multiple Wnt pathways and contribute to tumorigenesis.

### Activation of Rac1 shifts Rac1–β-catenin complexes from the membrane to nucleus

In order to understand how Rac1 boosts Wnt signaling we used a proximity ligation assay (PLA) coupled with confocal microscopy to image the localization patterns of hundreds of endogenous Rac1–β-catenin complexes throughout the cell. In untreated cells, Rac1–β-catenin complexes were mainly located at the plasma membrane, which was unsurprising given their roles in cell adhesion and migration (Citi et al., 2011; Valenta et al., 2012). Wnt stimulation elicited a shift of Rac1–β-catenin complexes away from the membrane towards the nucleus. A similar observation was made using an antibody specific for the active form of endogenous Rac1. Consistent with this, analysis of ectopic forms of Rac1 revealed that inactive Rac1–β-catenin complexes were mostly at the plasma membrane, whereas the active Rac1–β-catenin complexes displayed a more nuclear localization. Together, these data suggest a partial shift in Rac1–β-catenin complexes towards the nucleus upon Rac1 activation and/or Wnt stimulation.

Interactions between active Rac1 and β-catenin have been previously reported in the nuclei of cells (Esufali et al., 2007; Buongiorno et al., 2008); however, this is the first detection of an interaction between inactive Rac1 (T17N) and β-catenin and evidence that the distribution is regulated. Upon addition of Wnt we observed both an increase in active Rac1–β-catenin complex number and a specific increase in the nucleus, but it is unclear whether this was due to Wnt activation of Rac1 as previously suggested (Habas et al., 2003; Malliri et al., 2006; Bikkavilli et al., 2008; Wu et al., 2008; Valls et al., 2012; Chahdi and Raufman, 2013) or via the stabilization and increased expression of β-catenin. These data highlight the possibility that Wnt not only stabilizes β-catenin but may also activate Rac1 itself or Rac1-GEFs to ensure nuclear persistence of active Rac1–β-catenin complexes.

### Rac1 activity regulates β-catenin–LEF-1 complex formation in the nucleus of cells

The overexpression of LEF-1 drives nuclear accumulation of wild-type β-catenin (Behrens et al., 1996; Huber et al., 1996; Henderson et al., 2002). Notably, the nuclear accumulation of Rac1-sensitive β-catenin point mutants (S191/S605A) was less responsive to LEF-1 expression (Fig. 6), which could explain functional data from the Long laboratory who observed a significant reduction in TOPflash activity for both point mutants when compared with wild-type β-catenin (Wu et al., 2008). The distribution of β-catenin-S605A was particularly immune to the presence of LEF-1, which might be explained by the region in which the post-translational modification resides. S605 lies within the armadillo (Arm) repeat 10–12 region of β-catenin which, in combination with the carboxy terminus, is known to mediate transactivation function (Valenta et al., 2012). Chibby and ICAT, two physiological inhibitors of β-catenin–LEF-1 interaction (Graham et al., 2002; Li et al., 2008) both bind within this region, whereas LEF-1 binds between S191 and S605 within the arm repeats 3–9 (Xu and Kimelman, 2007). We speculate that addition of the phosphate group at S605 might disturb interaction between β-catenin and the inhibitors, but retains or enhances β-catenin interaction with LEF-1 and recruitment of transcriptional co-activators such as BCL9, pygopus, Mediator and CBP (Mosimann et al., 2009).

### Implications for cancer

Due to the tumor-promoting and cell migration-inducing activities of Rac1 and accumulated β-catenin in colon cancer (Fritz et al., 1999; Gómez del Pulgar et al., 2007; Jamieson et al., 2012; Jamieson et al., 2014), it is crucial to gain a further understanding of the signaling pathways and underlying mechanisms that contribute to Wnt transactivation. Our model offers a novel role for active Rac1 in modulating the Wnt signaling pathway and provides an alternative explanation for the often-reported links between Rac1 expression and Wnt in relation to colorectal cancer (Fritz et al., 1999; Gómez del Pulgar et al., 2005; Polakis, 2012) and colorectal carcinoma cell lines including Lovo, SW480, SW620, SW116 and HT-29 (Zhao et al., 2009). The development of small molecule inhibitors targeting β-catenin–LEF-1 interactions have been met with specificity difficulties due to an overlapping binding region between LEF-1/TCFs and destruction complex members such as APC and Axin (Jamieson et al., 2014). Thus further understanding of the mechanism by which Rac1 modulates the interaction between LEF-1 and β-catenin may provide an alternative therapeutic avenue to reduce β-catenin–LEF-1 signaling in colorectal cancer.

## MATERIALS AND METHODS

### Plasmids, siRNAs and antibodies

The following plasmids were transfected into cells: pmyc-β-catenin-WT, pmyc-β-catenin-S191A, pmyc-β-catenin-S191D, pmyc-β-catenin-S605A, pmyc-β-catenin-S605D, pEGFP-N1, pGFP-Rac1-WT, pGFP-Rac1-T17N and pGFP-Rac1-Q61L. The Rac1-sensitive β-catenin myc-tagged phosphorylation mutants were a kind gift from the Long laboratory (Division of Endocrinology, Metabolism and Lipid Research, Washington University in St Louis, USA) (Wu et al., 2008) and the GFP-tagged Rac1 constructs were a kind gift from the Philips laboratory [New York University (NYU) Cancer Institute, NYU School of Medicine NY, USA] (Michaelson et al., 2008). Cloning of the plasmid pβ-catenin-WT-GFP was previously described (Sharma and Henderson, 2007), and the mutant form pβ-catenin-S605A-GFP was made by PCR-amplifying the cDNA insert of pmyc-β-catenin-S605A and cloning in-frame into the KpnI and BamH1 sites of pEGFP-N1. The S605A mutation was verified by sequencing. The small interfering RNA (siRNA) targeting Rac1 was a pool of three target specific siRNAs (sc-44325, Santa Cruz Biotechnology, USA). The following antibodies were used: rabbit polyclonal β-catenin (sc-7199, Santa Cruz Biotechnology), rabbit polyclonal Topoisomerase II (sc-13058, Santa Cruz Biotechnology), rabbit polyclonal myc (sc-789, Santa Cruz Biotechnology), mouse monoclonal HA (sc-7392, Santa Cruz Biotechnology), rabbit polyclonal HA (sc-805, Santa Cruz Biotechnology) mouse monoclonal β-catenin (610154, BD Transduction Laboratories, USA), mouse monoclonal total-Rac1 (610650, BD Transduction Laboratories; clone 23A8, Millipore, USA), mouse monoclonal active-Rac1 (26903, New East Biosciences, USA), mouse monoclonal β-tubulin (T0198, Sigma-Aldrich, USA), rabbit polyclonal GFP (A11122, Invitrogen, USA), mouse monoclonal LEF-1 (H00051176-M01, Abnova, Taiwan), Alexa-Fluor 488/594/405 (Invitrogen) and Hoechst (H6024, Sigma-Aldrich).

### Cell culture, drug treatment and transfection

SW480 and HCT116 human colon cancer epithelial cells, NIH 3T3 mouse fibroblasts and HEK 293 embryonic kidney cells were cultured in Dulbecco's modified Eagle's medium (DMEM) supplemented with 10% fetal bovine serum (FBS) and antibiotics (penicillin and streptomycin) at 37°C in a 5% CO_2_-humidified incubator. LiCl (203637, Sigma-Aldrich) was dissolved in water and made fresh at each use. NSC23766 (sc-204823, Santa Cruz Biotechnology) or EHT1864 (sc-361175, Santa Cruz Biotechnology) were dissolved in water and stored at −20°C in aliquots to avoid repeated freeze–thaw cycles. Drugs were diluted to the required concentration in media immediately prior to administration. The optimal non-toxic and effective dose was determined for each drug by titration, starting with functional concentrations reported in the literature. For transfection, cells were seeded onto coverslips or in 8-well chamber slides 24 h prior to transfection. Transfections were carried out using Lipofectamine 2000 (11668-019, Invitrogen) according to manufacturer's instructions and media were replaced 6 h post-transfection and incubated for a total of 48 h.

### Preparation of L/Wnt3a conditioned media

For the preparation of L and Wnt3a media, cells were seeded into T150 flasks (1:10 dilution) with complete DMEM. Cells were incubated at 37°C and the first batch of media was harvested after 4 days. Fresh DMEM was added to the cells and the second batch of media was harvested after 3 days. The first and second batches of harvested media was combined, filtered and stored at −20°C.

### Transcriptional reporter assay

HEK293T cells were transiently co-transfected with GFP-tagged Rac1 constructs (WT or Q61L) and β-catenin–TCF luciferase reporter constructs, TOPflash, which contains multiple optimal TCF/LEF-1 binding sites that induce transcription of a luciferase reporter gene when activated by β-catenin, or a negative control FOPflash, which contains mutant and inactivated TCF/LEF-1 binding sites. Forty-eight hours after transfection, cells were treated with 40 mM LiCl for 6 h prior to harvesting. Cells were then washed with 1× PBS and lysed with 50 µl luciferase buffer (E4030, Promega, USA). Lysates were collected after centrifugation (13,000 r.p.m. for 1 min) and assayed for luciferase activity according to manufacturer's instructions (E4030, Promega). The luciferase activities were normalized against the levels of GFP expression and presented as a TOPflash/FOPflash ratio. Each experiment was performed in duplicate and repeated three times.

### Quantitative real-time reverse transcription PCR

HEK293T cells were transfected with GFP alone or Rac1-WT-GFP and treated with Wnt3a conditioned media (6 h) before being sorted into a GFP-rich pool using fluorescence-activated cell sorting (FACS). Total RNA was extracted using the RNAeasy mini kit (74104, Qiagen, Germany), quantified by UV spectroscopy and the integrity was confirmed by gel electrophoresis. One microgram of RNA was reverse transcribed using the RT^2^ First Strand Kit (330401, Qiagen) according to manufacturer's instructions. Gene transcripts were quantified by real-time (RT) PCR using RT^2^ SYBR Green (330522, Qiagen) and GAPDH was used as a control. The following primer sets were used: human β-catenin forward primer 5′-CAGAAGCTATTGAAGCTGAGG-3′ and the reverse primer 5′-TTCCATCATGGGGTCCATAC-3′; human c-myc forward primer 5′-CTTCTCTCCGTCCTCGGAT- TCT-3′ and the reverse primer 5′-GAAGGTGATCCAGACTCTGACCTT-3′; human Axin2 forward primer 5′-ACTGCCCACACGATAAGGAG-3′ and the reverse primer 5′-CTGGCTATGTCTTTGGACCA-3′; human Cyclin D1 forward primer 5′-AAGTGCGAGGAGGAGGTCTT-3′ and the reverse primer 5′-GGATGGAGTTGTCGGTGTAGA-3′; human LEF-1 forward primer 5′-CTTTATCCAGGCTGGTCTGC-3′ and the reverse primer 5′-TCGTTTTCCACCATGTTTCA-3′; human GAPDH forward primer 5′-GAGTCAACGGATTTGGTCGT-3′ and the reverse primer 5′-GACAAGCTTCCCGTTCTCAG-3′.

All RT-PCR experiments were performed in triplicate and repeated twice on a Stratagene Mx3000 qPCR system (Agilent Technologies, USA).

### Western blotting and subcellular fractionation

SW480 cells treated with 50 µM NSC23766 for 6 or 24 h or SW480 cells 72 h after siCONTROL or siRAC1 transfection were harvested and washed with PBS. The nuclear and cytoplasmic fractions were extracted using NE-PER Nuclear and Cytoplasmic Extraction Reagents (78833, ThermoFisher Scientific, USA) according to the manufacturer's manual. Total lysates were prepared using Pierce RIPA buffer (89901, ThermoFisher Scientific) according to manufacturer's protocol. Samples were quantified by Bradford analysis and measured at 595 nm with a microplate reader. Normalized amounts of the samples were denatured in SDS sample buffer for 10 min at 95°C before being separated by SDS-PAGE and blotted onto polyvinylidene (PVDF) membranes (Millipore). Membranes were blocked with 5% non-fat dry milk-TBS before incubating with primary antibodies at room temperature for 2 h. Topoisomerase II and β-tubulin antibodies were used as nuclear-cytoplasmic loading controls, respectively. After being washed with 0.1% Tris-buffered saline and Tween 20 (TBST), membranes were incubated with anti-mouse/rabbit horseradish peroxidase conjugated (HRP) secondary antibodies (Sigma-Aldrich) for 1 h at room temperature. Western Bright ECL Western Blotting Kit (Advansta, USA) was used to detect chemiluminescence, and blots were imaged using the ChemiDoc MP Imaging system (Bio-Rad, USA).

### Immunoprecipitation

At 48 h post-transfection, NIH 3T3 cells or HEK293 cells were harvested and lysed in Pierce IP lysis buffer (87787, ThermoFisher Scientific). The lysates were centrifuged for 10 min at 13,000 r.p.m. at 4°C. One milligram of lysate was incubated with specific antibodies [e.g. anti-HA monoclonal antibody (sc-7392, Santa Cruz Biotechnology) or mouse IgG (I5381, Sigma-Aldrich)] overnight at 4°C with continuous end-over-end mixing. Thirty microliters of protein-G–agarose (11719416001, Roche, Switzerland) beads were added to the samples and incubated for a further 2 h at 4°C with continuous end-over-end mixing. Samples were then washed once with Pierce IP buffer and then with Tris-buffered saline (TBS) before being denatured in SDS sample buffer for 7 min at 95°C. Proteins were separated by SDS-PAGE and blotted onto nitrocellulose membranes (HATF00010, Millipore). Membranes were blocked with 5% non-fat dry milk for 1 h and then incubated with anti-myc (sc-789, Santa Cruz Biotechnology), anti-HA (sc-805, Santa Cruz Biotechnology) or anti-GFP (Invitrogen) polyclonal antibodies for 2 h at room temperature. After being washed three times in 0.1% TBST, membranes were incubated for 1 h at room temperature with anti-rabbit HRP secondary antibody (A9169, Sigma-Aldrich). Results were visualized by the Chemidoc MP imaging system (Bio-Rad).

### Immunofluorescence microscopy and FRAP assays

Cells were washed three times with phosphate-buffered saline (PBS), fixed with 3.7% Formalin-PBS (F1635, Sigma-Aldrich) then permeabilized with 0.2% Triton-X-100-PBS (0694, Amresco, USA) for 10 min, blocked with 3% BSA-PBS (A7906, Sigma-Aldrich) and incubated with primary antibodies for 1 h at room temperature. Cells were washed twice with PBS before secondary antibodies were added for 40 min. Cells were subsequently mounted with Vectashield (Vector Laboratories, USA) and visualized by fluorescence microscopy and imaged and scored using an Olympus FV1000 confocal laser microscope. Fluorescence recovery after photobleaching (FRAP) assay to measure nuclear import rates of β-catenin-GFP in live NIH3T3 cells was as described previously (Jamieson et al., 2011), using an Olympus FV1000 confocal laser microscope. Briefly, three pre-bleach images of the cell were acquired, then the nucleus was bleached (60–70 frames). Post-bleach imaging was in two stages: 30 frames at the fastest interval, then 30 frames at 10 s intervals. Fluorescence intensities for cytoplasm, nucleus and background were acquired using Olympus Fluoview software and exported to a Microsoft Excel file.

### Data analysis

Background values were subtracted from cytoplasmic and nuclear fluorescence intensities, which were then expressed as a nuclear/cytoplasmic ratio. For each cell data set, the pre-bleach ratio was set to 100%, the time for the first post-bleach image was set to 0 s, and the recovery curve was adjusted to start at 25% recovery at time 0 (the closest average value). The average of the data from two experiments was plotted. Initial nuclear import rates for the first 30 s were analyzed with GraphPad Prism 5.0 software using linear regression analysis.

### Duolink proximity ligation assay

Cells cultivated for 48 h in 8-well chamber slides (Lab Nunc, ThermoFisher Scientific) were fixed with 3.7% formalin in PBS for 30 min before being permeabilized with 0.2% Triton-X-100 in PBS for 10 min. Cells were then subjected to the PLA assay using the Duolink red kit (O-link Bioscience, Sweden) in a humidity chamber according to the manufacturer's instructions. Briefly, plastic wells were removed from the chamber slides prior to blocking and incubation with primary antibodies and PLA probes (containing oligonucleotides) for 1 h. Ligase was then added to hybridize PLA probes and generate a closed circle before DNA amplification and conjugation to Alexa Fluor 594 for 100 min. After the PLA protocol, cells were then counterstained with anti-β-catenin mAb, myc pAb or HA mAb and Alexa Fluor 488 or 405 to visualize β-catenin and HA staining. Cells were then mounted onto coverslips with Duolink mounting media with 4′,6-diamidino-2-phenylindole (DAPI; O-link Bioscience) or Vectashield (Vector Laboratories, USA). *z*-stack images were taken using an Olympus FV1000 confocal laser scanning microscope with 60× water objective at 0.2 µm. Over 100 cells were imaged for analysis and each red puncta within the nuclei of cells was scored as a single interaction site.

### Statistics

Data analysis was performed using Statview (verison 5, SAS Institute, USA) or GraphPad Prism 5 software by one-way ANOVA or Student's *t*-test. Differences were considered to be statistically significant when *P*<0.05.
